# Fatal hepatitis E viral infection in pregnant women in Ghana: a case series

**DOI:** 10.1186/1756-0500-5-478

**Published:** 2012-09-03

**Authors:** Joseph Humphrey Kofi Bonney, Robert A Kwame-Aryee, Samuel Obed, Ama Asantewa Tamatey, Jacob Samson Barnor, Naa Baake Armah, Samuel Antwi Oppong, Mubarak Osei-Kwesi

**Affiliations:** 1 Noguchi Memorial Institute for Medical Research, School of health Sciences, University of Ghana, Accra, Ghana; 2 Department of Obstetrics and Gynaecology, Korle-Bu Teaching Hospital, P.O. Box KB 82, Accra, Ghana

**Keywords:** Fulminant hepatitis, Fulminant hepatic failure, Hepatitis E virus

## Abstract

**Background:**

Viral infections during pregnancy can pose serious threats to mother and fetus from the time of conception to the time of delivery. These lead to congenital defects, spontaneous abortion and even death. The definitive diagnosis and management of pregnancy-related viral infections may be challenging especially in less resourced countries.

**Case presentation:**

We present clinical and laboratory responses to the diagnosis and management of three cases of fulminant hepatitis secondary to Hepatitis E viral infection in pregnancy.

Case 1 was a 31-year-old Ghanaian woman who presented with a week’s history of passing dark urine as well as yellowish discoloration of the eyes. She subsequently developed fulminant hepatitis secondary to Hepatitis E viral infection, spontaneously aborted at 24 weeks of gestation and later died.

Case 2 was also a 31-year-old Ghanaian woman who was admitted with a four-day history of jaundice. She had low grade fever, but no history of abdominal pain, haematuria, pale stool or pruritus. She next developed fulminant hepatitis secondary to Hepatitis E viral infection. However, she did not miscarry but died at 28 weeks of gestation.

Case 3 was a 17-year-old Ghanaian woman who was referred to the tertiary health facility on account of jaundice and anaemia. She had delivered a live male infant at maturity of 32 weeks but noticed she was jaundiced and had a presentation of active disease 3 days prior to delivery. The baby was icteric at birth and on evaluation, had elevated bilirubin (mixed type) with normal liver enzymes. Hepatitis E virus infection was confirmed in both mother and baby. However, the jaundice and the hepatomegaly resolved in mother and baby after 5 and 12 days respectively.

**Conclusion:**

To the best of our knowledge, these are the first documented cases of fatal fulminant hepatic failures resulting from HEV infection in Ghana.

## Background

Hepatitis E virus (HEV) infection is a recognised cause of human viral disease presenting as an acute hepatitis after an incubation period of 8-10 weeks with a clinical illness resembling other forms of acute viral hepatitis [1]. Transmission is primarily through the faeco-oral route; usually from contaminated water. The disease is usually self-limiting and has a low case-fatality rate 0.1% in men and non-pregnant women [1]. The mortality rate is however severe, often leading to fulminant hepatic failure (FHF) and death in up to 30% of cases in HEV-infected pregnant women [2].

The seroprevalence of HEV infection amongst pregnant women in Accra, Ghana, has been found to be as high as 29% [3]. There is no or limited screening for anti-HEV antibodies for pregnant women during routine reproductive health programmes in Ghana. Public and most private health institutions however screen for antibodies to hepatitis B virus (HBV) and hepatitis C virus (HCV) in pregnant women on suspicion of viral hepatitis in Ghana [3]. Therefore the diagnosis of HEV infection in pregnant women could be delayed or misdiagnosed with a possible fatal outcome if the disease runs a fulminant course.

These three cases which resulted in two fatalities highlight the preponderance of HEV in a less-resourced country and the devastating effect it poses to pregnant woman.

The aim of the present case series is to present our experience with FHF in three patients who had active disease in Ghana and also facilitate the urgency in the establishment of appropriate routine laboratory diagnosis to enhance treatment.

## Case presentation

### Case 1

A 31 year old primigravida at 24 weeks gestation who was admitted in July, 2010 with over a week’s history of passing dark urine and yellowish discoloration of the eyes. She had developed fever (temperature >38°C), chills, headache and weakness by the time of her admission and was thought to have intravascular haemolysis of unknown aetiology. She had been treated for laboratory confirmed malaria at 10 weeks and subsequently started on intermittent preventive treatment. Fever (temperature >38°C) persisted, became anaemic (Hb of 8.7 g/dL) and jaundiced and started passing dark urine. No definite cause was identified after various investigations were done: blood film was negative for malaria parasites. Her sickling status was negative and she was negative for HBV and HCV. Glucose-6-phosphate dehydrogenase (G6PD) levels were normal. The disease progressively worsened; became very lethargic, deeply jaundiced and febrile and developed bilateral pitting pedal oedema up to the knee level. A differential diagnosis of haemorrhagic fever was entertained after she developed sub-conjunctival haemorrhages. Further laboratory investigations were performed. She had a miscarriage after the second day of admission at 24 weeks gestation and died on the third day.

Laboratory investigations for molecular testing with Reverse transcription – Polymerase chain reaction (RT-PCR) assay, specific for yellow fever virus [4] performed were negative. Further molecular analysis performed with an assay based on the publication; ‘A novel real-time PCR system for simultaneous detection of human viruses in clinical samples with uncertain aetiology’ [5] revealed a positive RNA for HEV infection.

The results of preliminary laboratory investigations done were: haemoglobin of 7.4 g/dL; white blood cell count - 4.0×10^9^per L; platelet count - 191×10^9^per and the erythrocyte sedimentation rate (ESR) was 28 mm/hr. A peripheral blood smear done for malaria parasites was negative. The Clotting profile showed an activated partial thromboplastin time (aPTT) of 132.0 seconds (27.0-43.0s) and INR of 3.63 (1.00-1.25). Liver function tests results were out of range (Table 1). The results for both HBV and HCV with the real time RT-PCR assay by Katano et al [5] were negative. Serological testing revealed no antibodies that are both immunoglobulin G (IgG) and immunoglobulin M (IgM) for viral hepatitis E, B and C.

**Table 1:** Liver function tests results

**Test**	**Patients value**				[14,15]** Ref. range (Unit)**
	**Case 1**	**Case 2**	**Case 3**		
			**Day 1**	**Day 12**	
Total Protein	61	61.3	68	73	**60 - 78 (g/L)**
Albumin	16	32.4	27	31	**35 - 55 (g/L)**
Globulin	27.6	28.9	-	-	**23 - 35 (g/L)**
Alkaline Phosphatase (ALP)	94	379	472	215	**42 - 98 (U/L)**
Gamma Glutamyl Transpeptidase (GGT)	46	100.7	17	22	**5 - 40 (U/L)**
Alanine Transaminase (ALT)	609	557	50	13	**7 - 56 (U/L)**
Aspartate Transaminase (AST)	1460	1327	80	21	**6 - 34 (IU/L)**
Direct (conjugated) Bilirubin	170	40.5	17	11.7	**0.0 - 5 (μmol/L)**

### Case 2

A 31-year-old gravid 5 para 3 with one previous termination of pregnancy was admitted with a 28 weeks old pregnancy and a four-day history of jaundice with low grade fever (37.7°C). She had no previous history of blood transfusion, jaundice or contact with a jaundiced patient. Clinical and biochemical examinations were performed. Obstetric and general abdominal ultrasounds showed a normal and viable intra-uterine fetus at 28 weeks, 4 days gestation. Laboratory results obtained showed normal haemogram, sickling test was negative and no malaria parasites were identified. Test performed for HBV and HCV revealed no anti-HBV or anti-HCV IgG and IgM antibodies. The liver function test (LFT) results were severely deranged (Table 1).

The patient’s clinical condition remained quite stable until she started spiking temperature on the fifth day of admission. The temperature was 39.0°C and examination revealed left renal angle tenderness. Two days into the parenteral antibiotic therapy the patient continued to spike temperature and the jaundice began to deepen with the production of dark urine. Further molecular and biochemical tests were performed. A positive result for HEV with the molecular test assay described above for case 1 was revealed.

After a week of admission the patient was found to be febrile (T = 39°C), tachypnoeic with respiratory rate of 60 cycles/minute and signs of consolidation of the left middle lung zones. Diagnosis was reviewed to include septicaemia secondary to left lobar pneumonia and urinary tract infection and possible severe malaria. The clotting profile showed INR of 4.2 and aPTT of 111.4 secs (reference range: 26-36 secs). She developed signs of hepatic encephalopathy and was started on hepatic failure regime. She was transfused with fresh frozen plasma in preparation for induction of labour, but she died on the eighth day before the labour induction could be started.

### Case 3

**Figure 1:**
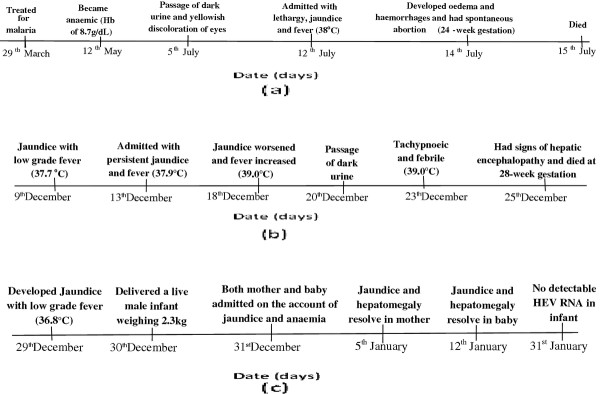
**Time series of the events by date of occurrence in the three case reports of fatal hepatitis E viral infection in pregnant women in Ghana: (a) case 1 was a 31-year-old woman with a week’s history of dark urine and yellowish discoloration of the eyes.** She was subsequently diagnosed of Hepatitis E viral infection, spontaneously aborted at 24 weeks of gestation and later died; (**b**) case 2, also a 31-year-old who was admitted with a four-day history of jaundice. She had low grade fever, but no history of abdominal pain, haematuria, pale stool or pruritus. Later diagnosed of Hepatitis E viral infection, did not miscarry but died at 28 weeks of gestation; (**c**) case 3, a 17-year-old woman who was referred to the tertiary health facility on account of jaundice and anaemia. She had delivered a live male infant at maturity of 32 weeks but noticed she was jaundiced and had a presentation of active disease 3 days prior to delivery. The baby was icteric at birth and hepatitis E virus infection was confirmed in both mother and baby. However, the jaundice and the hepatomegaly resolved in mother and baby after 5 and 12 days respectively.

A 17-year-old primipara was admitted on account of jaundice and anaemia. She had delivered a live male infant weighing 2.3 kg at a maturity of 32 weeks, a day before. The baby was icteric at birth and on evaluation, had elevated bilirubin (mixed type) with normal liver enzymes. She had noticed the jaundice 3 days prior to delivery, but it worsened after the delivery and she therefore reported back to the medical facility. On direct questioning she had a low grade fever, chills and had vomited once at the onset of the jaundice. There was no change in her bowel habits or the colour of her urine. There was no history of transfusion or contact with a jaundiced person. On examination her temperature was 36.8°C, chest clinically clear and no abnormality with her cardiovascular system; the blood pressure was 90/50 mmHg. The liver was enlarged, 5 cm below the costal margin with a span of 15 cm, but not tender. There was no ascites. The spleen and two kidneys were not palpable. A diagnosis of acute viral hepatitis and malaria with intravascular haemalysis were made for both mother and baby and were admitted separately. HEV infection was confirmed in both mother and baby after admission with the molecular assay used in the two cases above. The jaundice and the hepatomegaly resolved in mother and baby after 5 and 12 days respectively. The HEV-infected baby had serial clinical, serological and virological evaluation. Liver tests settled to normal levels by 8 weeks. HEV RNA which was detected at birth was not detectable by 3 weeks. IgM anti-HEV antibodies, reactive at birth persisted for 4 weeks but did not lose IgG anti-HEV antibodies over the observation period of three months (Figure 1).

## Discussion

To our knowledge, these are the first documented cases of fatal fulminant hepatic failures (FHF) resulting from HEV infection in Ghana. Hepatitis E virus infection had hitherto been an unknown cause of jaundice in Ghana.

The first case had complained of general malaise and pruritus about 8 weeks prior to developing clinical jaundice. Platelet count was in the normal range and her erythrocyte sedimentation rate (ESR), a non-specific measure of inflammation, was relatively high. The ratio of AST/ALT used sometimes in differentiating the causes of liver damage [6] was greater than 2. This is usually indicative of alcoholic hepatitis [7,8] and not viral hepatitis, in which case the ratio would have been less than 1. Case one neither drank alcohol nor smoked cigarettes. Liver function tests done at the time showed mild elevation of direct bilirubin and liver transaminases. This early elevation in bilirubin probably suggested HEV infection since physiological jaundice developed later. In a study by Patra, S. et al., it was found that death from FHF has been seen to be more common with HEV-infected pregnant women, or have worse foetal outcomes than did pregnant women with jaundice and acute viral hepatitis caused by other hepatitis viruses [9]. This finding is consistent with our observation in the first two cases. Furthermore, it has been established that HEV-infected pregnant women in the second and third trimester develop FHF with the mortality in the second trimester around 20% and reaches up to 45% in the third trimester [10]. This finding corroborates with our observation in that the first two fatal cases were HEV-infected pregnant women in their second trimester.

It has been previously reported that a high proportion of HEV-infected pregnant women develop FHF which is mostly fatal as we observed with these three cases. In a report of an HEV epidemic, 22% of pregnant women with acute viral hepatitis developed FHF, compared with 0% of non-pregnant women and 2.8% of men [10]. Other studies documented the development of FHF in 32% to 69% of HEV-infected pregnant women with acute viral hepatitis in sporadic settings [11]. Reasons that seem likely for the high frequency and severity of FHF in HEV-infected pregnant women are the diminished cellular immunity and high levels of steroid hormones which influence hepatitis E virus replication during pregnancy [12].

In our third case vertical transmission of HEV was observed. Vertically transmitted HEV infection is known to cause acute hepatitis in neonates but the clinical course and duration of viraemia is not known [13]. The survival and the clearance of HEV in the baby in our third case demonstrate that HEV in neonates is self-limiting and does not run a chronic or prolong clinical course. This observation is consistent with a study by Khuroo, M.S et al., in which HEV-infected neonates at birth survived with the infection cleared [13]. The severity of HEV infection in the mother and baby may be linked to each other. Data from a study suggested that foetal disease influenced the course of maternal HEV infection and provides a clear relationship between FHF of the fetus and mother [14].

Routine laboratory investigations for HEV and other hepatotropic viruses apart from HBV and HCV in health institutions in less-resourced countries has been a challenge. There is a likelihood of missing out on or misdiagnosing such cases in health institutions in less-resourced countries where there is low index of suspicion for HEV. Usually in such instances, Yellow Fever virus, which is also a hepatotropic virus, is suspected and queried as in our first case. The diagnosis of the second and the third cases were facilitated by the knowledge of the existence of fulminant HEV infection in the country and where to get diagnosis having excluded HBV and HCV infections.

HEV infection is considered an important public health concern in most developing and industrialized countries for causing water borne outbreaks and sporadic hepatitis because of poor sanitary conditions. There is no specific treatment available for Hepatitis E Virus infection. Recombinant vaccines are being developed and may be particularly useful for travellers to disease-endemic areas and for pregnant women [15]. Ensuring a clean drinking water supply remains the best preventive strategy, however, incorporating screening for HEV in reproductive health programmes could be vital for early detection and reduction of maternal mortality due to acute viral hepatitis.

## Conclusion

Although the cases reported here are few, two deaths in three HEV infections in pregnancy are very high (case fatality rate of 67%). Further investigations from other parts of the country and other places of similar settings are necessary to generate data which will guide the formulation of appropriate public health policy on HEV in less-resourced countries.

## Consent

### Case 1 and 2

Written informed consent was obtained from the patients’ next of kin for publication of this manuscript. A copy of the written consent is available for review by the Editor-in-Chief of this journal.

### Case 3

Written informed consent was obtained from the patient’s legal guardian for publication of this manuscript. A copy of the written consent is available for review by the Editor-in-Chief of this journal.

Consent was sought from the guardians of the death patients and the patient who survived.

## Competing interests

The authors declare that they have no competing interests.

## Authors’ contributions

JHKB participated in laboratory investigation and the manuscript writing and editing, RAKA was involved in the healthcare of the patients, writing and editing of the manuscript, SO was involved in the healthcare of the patients, AAT was involved in the healthcare of the patients, writing and of the manuscript, JSB was involved in the editing of the manuscript, NBA was involved in the healthcare of the patients, writing and editing of the manuscript, SAO was involved in the healthcare of the patients, MOK supervised the design and implementation of the study protocol. All authors read and approved the final manuscript.
